# A split luciferase-based probe for quantitative proximal determination of Gα_q_ signalling in live cells

**DOI:** 10.1038/s41598-018-35615-w

**Published:** 2018-11-21

**Authors:** Timo Littmann, Takeaki Ozawa, Carsten Hoffmann, Armin Buschauer, Günther Bernhardt

**Affiliations:** 1 Institute of Pharmacy, University of Regensburg, Universitätsstraße 31, D-93053 Regensburg, Germany; 2 Department of Chemistry, School of Science, University of Tokyo, 7-3-1 Hongo, Bunkyo-ku, Tokyo 113-0033 Japan; 3 Institute of Molecular Cell Biology, University Hospital Jena, University of Jena, Hans-Knöll-Str. 2, D-07745 Jena, Germany

## Abstract

The earlier an activation of a G protein-dependent signalling cascade at a G protein-coupled receptor (GPCR) is probed, the less amplificatory effects contribute to the measured signal. This is especially useful in case of a precise quantification of agonist efficacies, and is of paramount importance, when determining agonist bias in relation to the β-arrestin pathway. As most canonical assays with medium to high throughput rely on the quantification of second messengers, and assays affording more proximal readouts are often limited in throughput, we developed a technique with a proximal readout and sufficiently high throughput that can be used in live cells. Split luciferase complementation (SLC) was applied to assess the interaction of Gα_q_ with its effector phospholipase C-β3. The resulting probe yielded an excellent Z’ value of 0.7 and offers a broad and easy applicability to various Gα_q_-coupling GPCRs (hH_1_R, hM_1,3,5_R, hNTS_1_R), expressed in HEK293T cells, allowing the functional characterisation of agonists and antagonists. Furthermore, the developed sensor enabled imaging of live cells by luminescence microscopy, as demonstrated for the hM_3_R. The versatile SLC-based probe is broadly applicable e.g. to the screening and the pharmacological characterisation of GPCR ligands as well as to molecular imaging.

## Introduction

G protein-coupled receptors (GPCR) consist of seven transmembrane helices and are responsible for transducing stimuli, e.g. by hormones or neurotransmitters, across the cellular membrane. They represent the largest of all protein superfamilies in the human genome comprising more than 1000 different receptors^1^, and are the most important drug targets with approximately 34% of all drugs addressing GPCRs^2^. Agonist binding to a GPCR leads to the activation of heterotrimeric G proteins comprising an α, β and γ subunit. Binding of an agonist to a GPCR leads to a structural rearrangement resulting in an exchange of GDP for GTP within the α subunit.

There are four major subfamilies of Gα proteins of which we focussed on the α_q_ type that upon activation of the receptor, interacts with effector proteins of the phospholipase C (PLC) class and triggers their enzymatic activity. PLCs catalyse the formation of inositol trisphosphate (IP_3_) and diacylglycerol (DAG) from phosphatidylinositol 4,5-bisphosphate. DAG diffuses in the cell membrane, whereas IP_3_ activates Ca^2+^ channels within the membrane of the endoplasmic reticulum and/or the cellular membrane, both leading to a transient increase in the concentration of Ca^2+^ in the cytosol. The latter is involved in a plethora of physiological processes such as rearrangements of the cytoskeleton and regulation of gene transcription^3^.

In case of the second messengers IP_3_ and Ca^2+^, changes in intracellular levels are usually measured by liquid-scintillation counting or luminometry. When cells are incubated with tritiated *myo*-inositol, radioactive IP_3_ (IP_1_, IP_2_) levels can be determined^4^, whereas Förster resonance energy transfer (FRET)^5^- and split-luciferase complementation (SLC)-based^6^ assays make use of specific interactions of IP_1_ or IP_3_ with various optical probes. Most often intracellular Ca^2+^ levels are measured either with fluorescent chelators, changing their optical properties upon complexation of Ca^2+^ ions^7–9^ or a calcium-dependent luciferase (aequorin)^7,10^.

However, substantial amplification, taking place with every step in the signalling cascade, potentially masking effects at earlier stages^11^ can lead to misinterpretation, e.g. partial agonists appear as a full agonists in assays with a distal readout^3,12^. Furthermore, since there is an increasing interest in the discrimination of biased agonism with respect to G protein-dependent and β-arrestin-dependent pathways^13^, techniques are needed, allowing a proximal quantification of signalling events^14^. Such methods comprise [^35^S]GTPγS-incorporation^15^ and steady-state [^32,33^P]-based GTPase assays^16^ or FRET^17^- and bioluminescence resonance energy transfer (BRET)-based techniques^18^.

Most of the aforementioned approaches are compromised e.g. by requiring cell lysis, the preparation of membranes, the availability of radiolabelled chemicals or by low throughput. To overcome these limitations, we decided to use SLC to quantify the interaction of Gα_q_ with PLC-β3. This technology is based on two catalytically inactive complementary fragments of a luciferase, reconstituting a functional enzyme, catalysing the oxidation of a substrate with concomitant emission of light, when brought in close proximity^19^. The two fragments are fused to two proteins of which a specific interaction is expected, in this case Gα_q_ and PLC-β3. Probes, based on SLC, have become valuable tools for the quantification of protein-protein interactions (PPI) in general^20–22^, but also in the field of GPCR research. In this context, SLC was successfully applied to probe the interaction of β-arrestins with GPCRs^23–25^ and to the quantification of second messengers such as cAMP^26^ and IP_3_^6,26^. Advantages of SLC involve a high signal-to-background (S/B) ratio, enabling live cell and *in vivo* imaging^27^ and the availability of luciferases catalysing chemical reactions, accompanied by the emission of bright light of different wavelengths (broad spectral diversity)^28–30^.

**Figure 1:**
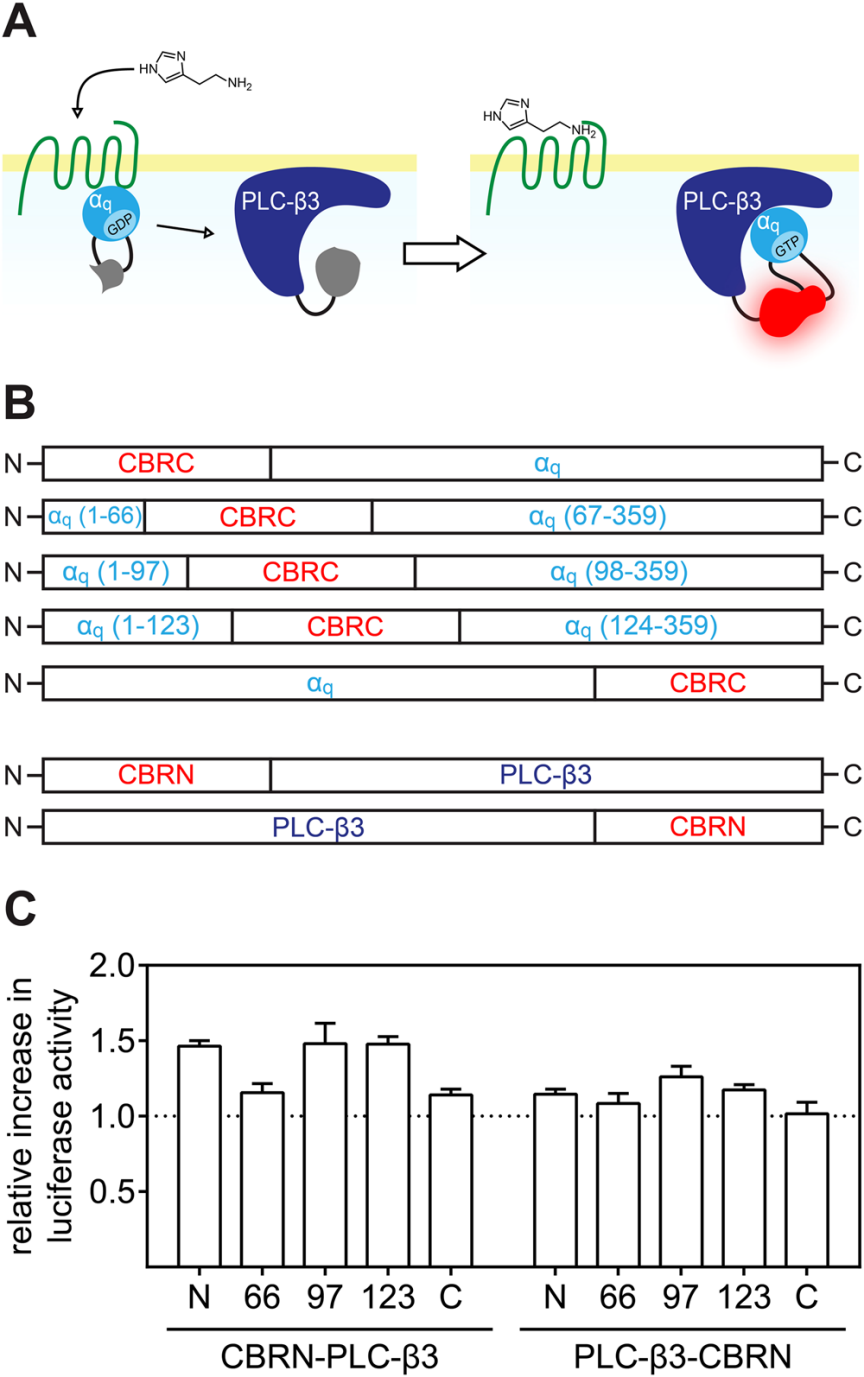
Schematic illustration of the sensor principle and the fusion protein library used to determine the best combination of proteins. The activation of the Gα_q_ pathway was probed by fusing complementary luciferase fragments to Gα_q_ and PLC-β3 (**A**). A fusion protein library was generated by fusing CBRC to Gα_q_ terminally and in three loop regions (numbers in parentheses denote amino acid positions) and by fusing CBRN either N-, or C-terminally to PLC-β3 (**B**). The different combinations of Gα_q_ and PLC-β3 fusion proteins were expressed in HEK293T cells, co-expressing the hH_1_R. The relative increase in luminescence of cells stimulated with 10 µM histamine compared to unstimulated cells is shown for each combination (**C**). Data are presented as means ± SEM from three independent experiments, each performed in triplicate.

We applied SLC to probe the Gα_q_/PLC-β3 interaction (Fig. 1A) by means of a modified luciferase from the click-beetle *Pyrophorus plagiophthalamus* (λ_max_ = 613 nm). The enzyme was split into two fragments, a larger N-terminal fragment (CBRN) consisting of the amino acids 1–416 and a smaller C-terminal fragment (CBRC) composed of amino acids 395–542. We generated two sets of fusion proteins of which the first one represents CBRN fused either N-, or C-terminally to PLC-β3 and in the second one CBRC was fused terminally to Gα_q_. As both termini of Gα subunits are known to be crucial not only for interactions with a respective GPCR the βγ-complex but also for the association with the cellular membrane^31–34^, CBRC was also integrated in three different flexible loop regions of Gα_q_. The combination of those fusion proteins, giving the highest S/B ratio upon complementation was used as a sensor to probe the activation of different Gα_q_-coupled receptors. We demonstrate that the new probe is of value for the functional characterisation of GPCR ligands and for imaging receptor activation in live cells.

## Material and Methods

### Materials

Dulbecco’s modified Eagle’s medium (DMEM) with and without phenol red and phosphate-buffered saline (PBS) were from Sigma (Germany). Leibovitz’ L-15 medium (L-15) and Hank’s balanced salt solution (HBSS) were from Gibco (Germany). Fura-2 AM, fetal calf serum (FCS), trypsin and geneticin (G418) were from Merck Biochrom (Germany). D-Luciferin was purchased as potassium salt either from Wako (Japan) or from Pierce (Germany) and was dissolved in HBSS at a concentration of 400 mM. Puromycin was obtained from Invivogen (France). The pCBR-control vector and the Bright-Glo luciferase assay reagent were from Promega (Japan and Germany). The pcDNA4 vector was from Thermo Scientific (Germany), whereas the pIRESpuro3 vector was from Clontech (France). Depending on their physicochemical properties, when possible, ligands were dissolved in H_2_O; otherwise DMSO (Merck) was used as solvent. Histamine dihydrochloride (his), was from Acros Organics (Belgium), betahistine dihydrochloride (betahis), diphenhydramine hydrochloride (diph), cyproheptadine hydrochloride (cyp), maprotiline hydrochloride (map), carbachol chloride (car), iperoxo iodide (iper), N-methyl scopolamine bromide (NMS), atropine (atr), propantheline bromide (prop) and pirenzepine dihydrochloride (pir) were from Sigma (Germany), whereas mepyramine maleate (mep) was from Tocris Bioscience (MO, USA). UR-KUM530 (KUM530)^35^ and histaprodifen (histapro) were kindly provided by Prof. Dr. Sigurd Elz (University of Regensburg, Germany). Oxotremorine sesquifumarate (oxo) was from MP Biomedicals (CA, USA), xanomeline (xan) was synthesized in-house according to a standard procedure^36^. Neurotensin (8–13) (NT(8-13)) was from Synpeptide (China). SR142948A was gift from Dr. Harald Hübner (University of Erlangen, Germany). FR900359 was purchased from the Institute of Pharmaceutical Biology, University of Bonn (Germany).

### Cell cultivation

In this study, the HEK293T cell line, obtained from the German collection of microorganisms and cell cultures (DSMZ), was used. Cells were routinely monitored for mycoplasma contamination using the Venor GeM Mycoplasma Detection Kit (Minerva Biolabs, Germany) and were negative. Unless otherwise stated, the cells were cultivated in DMEM containing 10% FCS (full medium) at 37 °C in a water-saturated atmosphere containing 5% CO_2_.

### Generation of plasmids

Plasmids encoding different human GPCRs were obtained from the Missouri cDNA resource center (MO, USA). The other plasmids used were generated by standard PCR and restriction techniques within the pcDNA backbone, unless otherwise stated. A plasmid encoding the red-emitting click-beetle luciferase (pCBR-control) was used as a template in different polymerase chain reactions (PCR) to generate the sequences encoding the two slightly overlapping N-terminal (*CBRN*, encoding amino acids 1–416) and C-terminal (*CBRC*, encoding amino acids 394–542) fragments of the luciferase. *CBRN* was then used to prepare plasmids encoding fusion proteins consisting of PLC-β3 fused either N- or C-terminally to CBRN. *CBRC* was used to generate plasmids of five different fusion proteins in which CBRC was fused to both termini of Gα_q_ or integrated into the Gα_q_ sequence after amino acids 66, 97 (Gα_q_(97)) and 123 (Gα_q_(123)), respectively. The linker sequences used to connect the luciferase fragments to either PLC-β3 or Gα_q_ consisted of flexible Gly and Ser residues. The cDNA encoding the Gα_q_(123) fusion protein and the N-terminally-tagged PLC-β3 were then subcloned into a pIRESpuro3 vector separated by a P2A autoproteolysis site^37^, yielding the pIRESpuro3 CBRN-PLC-β3-2A-Gα_q_(123) vector. Cleavage of the P2A site was controlled by immunoblotting (Fig. S2). All plasmids were quality controlled by means of enzyme restriction analysis and sequencing.

### Identification of the best pair of Gα_q_ and PLC-β3 fusion proteins

HEK293T cells were seeded on a 6-well plate (Sarstedt, Germany) at a density of 7 · 10^5^ cell/well. The next day, the cells were transfected with plasmids encoding the different combinations of fusion proteins and the human histamine H_1_ receptor (hH_1_R). After 48 h of incubation, the cells were detached by trypsinization, centrifuged and resuspended in DMEM devoid of phenol red, supplemented with FCS (5%). The concentration was adjusted to 1.11 · 10^6^ cells/mL; 90 µL of this suspension were seeded into each well of a white 96-well plate (Greiner Bio One, Germany), and the cells were incubated overnight. To induce interaction of the two fusion proteins, histamine was added at a concentration of 10 µM to the cells. In a control experiment, only DMEM without phenol red, the vehicle of histamine, was applied. After 25 min, 50 µL of medium were aspirated and replaced by 50 µL of Bright-Glo luciferase reagent. The cells were vigorously shaken for 2 min before luminescence was detected using a Genios Pro plate reader (Tecan) for 1 s per well.

### Generation of stable expression cell lines

HEK293T cells were transfected with the pIRESpuro3 CBRN-PLC-β3-2A-Gα_q_(123) vector as described above. After two days of incubation, the cells were detached using trypsin and were seeded into a 75-cm^2^ cell culture flask. Then, the cells were allowed to attach and puromycin was added at a concentration of 0.75 µg/mL. The cells were cultured upon changing the medium at regular intervals until stable growth was observed again. Subsequently, plasmids encoding cDNAs of GPCRs were transfected in the same way, with the exception that selection was achieved in the presence of geneticin.

### Quantification of agonistic potencies and antagonistic activities using the developed probe

Cells, expressing the developed Gα_q_/PLC-β3 sensor in combination with one of the GPCRs, were detached from a 75-cm^2^ flask by trypsinization and centrifuged (700 g for 5 min). The pellet was resuspended in assay medium consisting of L-15 with 5% FCS and the density of the suspension was adjusted to 1.25 · 10^6^ cells/mL. Then, 80 µL of this suspension were seeded into each well of a 96-well plate, and the plate was incubated at 37 °C in a humidified atmosphere (without additional CO_2_) overnight. On the next day, 10 µL of 10 mM D-luciferin (Pierce) were added to the cells, and the plate was transferred into a pre-warmed microplate luminescence reader (either a Genios Pro (Tecan, Austria) or an EnSpire (Perkin-Elmer, Germany)). The cells were allowed to equilibrate inside the reader for 10 min, before the basal luminescence was determined, by recording the luminescence for the entire plate ten times with an integration time of 1 s per well. In the meantime, serial dilutions of agonists were prepared, the resulting solutions were also pre-warmed to 37 °C and subsequently added to the cells. Thereafter, luminescence was recorded for 30 plate repeats amounting to a time period of 50 min. Negative controls (solvent) and positive controls (reference full agonist, histamine (hH_1_R), carbachol (hM_1,5_R), oxotremorine (hM_3_R)) eliciting a maximal response (100%) were included for subsequent normalization of the data. In case of the antagonist mode, antagonists were added 15 min prior to the initial thermal equilibration period to ensure an equilibrium between antagonists and receptors, before agonists were added. The pK_b_-values of antagonists were determined according to the Cheng-Prusoff equation^38^. FR900359 was pre-incubated for 20 min, before cells were stimulated with agonists. After acquisition of the data, the peak luminescence intensities obtained after stimulation were used for quantitative analysis using Prism 5 (Graph Pad).

### Fura-2 Ca^2+^ assay

HEK293T cells expressing either the hH_1_R alone, or co-expressing the Gα_q_/PLC-β3 sensor, were incubated with Fura-2 AM analysed in cuvettes using a LS50 B luminescence spectrophotometer (Perkin-Elmer, Germany). Fura-2 calcium assays were essentially performed as described previously^39^.

### Live cell luminescence microscopy

HEK293T cells, expressing hM_3_R and the Gα_q_/PLC-β3 sensor, were seeded on a 35-mm cell culture dish (Iwaki, Japan) in full medium at a density of 10^6^ cells/dish and were incubated overnight. The next day, 30 µL of 1 M HEPES buffer (pH 7.4) and 7.5 µL of 400 mM D-luciferin (Wako) were added. The cells were transferred to a IX-81 microscope (Olympus, Japan), equipped with a super-cooled EM-CCD camera (Hamamatsu photonics, Japan), with its stage heated to 37 °C, and bioluminescence microscopy was performed essentially as described^25^. Briefly, the images were acquired with an exposure time of 5 min per frame. After the first frame, oxotremorine was added to a final concentration of 100 nM. In case of the antagonist mode, atropine was added to a final concentration of 100 nM prior to the very first frame.

## Results and Discussion

### Development of the Gα_q_ activation sensor

In a conventional approach to develop a SLC-based PPI probe, a set of fusion proteins, comprising the luciferase fragments and the two host proteins is engineered and expressed. Usually, both luciferase fragments are fused to both termini of the host proteins and all combinations of the resulting fusion proteins are analysed with respect to their ability to restore the luminescence signal. As our aim was to probe the Gα_q_/PLC-β3 interaction (Fig. 1A), and because Gα_q_ is a rather small protein of which it is well known that both termini are of major importance for interactions with βγ-subunits, with a GPCR and the association with the cell membrane^31–34^, we pursued a slightly different strategy: we fused the smaller luciferase fragment (CBRC) to Gα_q_ and incorporated CBRC into three different flexible loop regions, localized within the helical domain of Gα_q_^40^ (Fig. 1B). The complementing part of the luciferase (CBRN) was fused either to the N-, or the C-terminus of PLC-β3 (Fig. 1B). To identify the best combination of fusion proteins in terms of luminescence intensity and S/B ratio, we expressed all combinations of Gα_q_ and PLC-β3 fusion proteins shown in Fig. 1B in HEK293T cells co-expressing the human histamine H_1_ receptor (hH_1_R). The resulting transfectants were stimulated with 10 µM histamine for 25 min before the cells were lysed and the substrate was added. The detected luminescence was normalized i.e. divided by the luminescence intensity emitted from unstimulated cells. The low normalized luminescence shown in Fig. 1C suggests that the C-terminus of PLC-β3 is rather far away from the interaction site with Gα_q_. This is supported by the crystal structure of the Gα_q_/PLC-β3 complex^40^. The CBRN-PLC-β3 fusion protein gave higher S/B ratios, especially when used in combination with the Gα_q_ variant, in which CBRC was incorporated after amino acid 97 or 123. However, the overall luminescence intensity was higher for Gα_q_(123) (Fig. S1).

Although the construct, in which CBRC was N-terminally fused to Gα_q_, showed higher luminescence (Fig. S1), but only an S/B ratio comparable to that of Gα_q_(97) and Gα_q_(123), respectively, this fusion protein was not considered, because Yu *et al*. reported that an N-terminal fusion of green fluorescent protein to Gα_s_ resulted in a lack of association with the cell membrane^34^. Therefore, we favoured Gα_q_(123) in combination with CBRN-PLC-β3.

We further optimized the sensor mainly with respect to handling. For this purpose, a vector plasmid, enabling convenient multi-cistronic expression of both fusion proteins in a fixed stoichiometry (1:1), using a self-cleaving P2A peptide sequence^37^, separating CBRN-PLC-β3 and Gα_q_(123), was constructed. Furthermore, we fabricated a HEK293T cell line, characterized by stable integration of the aforementioned plasmid into the genome as a versatile platform for the analysis of different GPCRs upon co-transfection. Cleavage of the P2A sequence was proven by immunoblotting using an anti-Gα_q_ antibody (Fig. S2). The western blots also revealed that the expression level of the modified Gα_q_ was similar to that of endogenous Gα_q_ in HEK293T cells (Fig. S2).

### Characterization of the new probe

**Figure 2:**
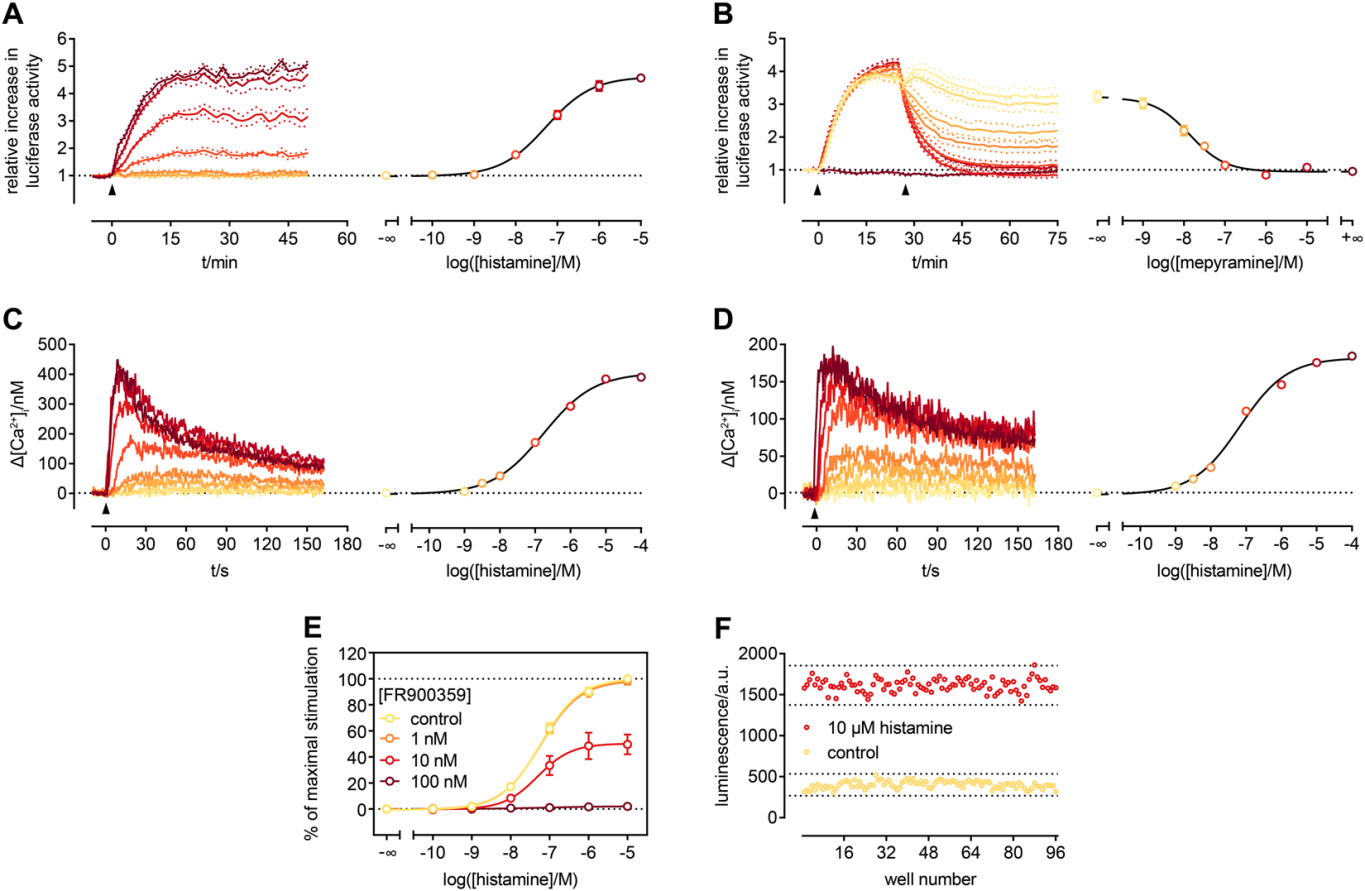
Characterization of the Gα_q_ activation sensor. All experiments were performed with HEK293T cells, expressing the Gα_q_ sensor and the hH_1_R, except for C were the sensor was not present. Increasing concentrations of histamine (addition indicated by arrow) lead to proportionally increasing luminescence emitted from the cells, which can be converted to a CRC (**A**). The opposite (a gradual decrease in luminescence) became obvious, when stimulated cells (300 nM histamine, first arrow) were subsequently treated with the selective hH_1_R agonist mepyramine (second arrow) (**B**). In case of the highest concentration, luminescence decreased to basal levels, indicating full reversibility of the sensor interaction. Since the observed activation kinetics in A was slower than expected for G protein activation, a kinetic Fura-2 assay for the quantification of [Ca^2+^]_i_ was performed, to guarantee that the sensor does not negatively influence downstream signalling. In cells, in which the sensor was present, the kinetics was the same (**C**) when compared to cells devoid of the sensor (**D**). The concentration-dependent response to histamine (addition indicated by arrow) was similar (hH_1_R alone: pEC_50_: 7.1 **±** 0.1; Gα_q_ sensor present: pEC_50_: 6.8 **±** 0.1), too. Furthermore, we were able to show that the modified Gα_q_ as part of the sensor was still prone to inhibition by FR900359 (**E**). The sensor shows an exceptionally good Z’ of 0.7 (**F**). Data in A-D are representative of at least two independent experiments. Data in E are presented as mean along with their SEM from five independent experiments performed in triplicate. Data in F was obtained from an entire 96-well plate.

To overcome the low S/B ratio, we added the substrate D-luciferin to live cells (in culture medium) rather than performing endpoint measurements after lysis of the cells. Thereby, we were able to follow the kinetics of the reaction (Fig. 2A). The sensor responded to an activation of the hH_1_R by increasing the concentration of histamine with a gradual increase in luminescence, which can be converted to a concentration-response-curve (CRC), yielding an EC_50_ value in very good agreement with data obtained from canonical assays^3,16^.

Additionally, we could show that the interaction of the two sensor proteins was fully reversible as demonstrated in Fig. 2B. The addition of mepyramine, an hH_1_R-specific antagonist, after activation of the receptor with histamine (300 nM) led to a concentration-dependent decrease in luminescence, down to the background level. Similar experiments were performed with a sensor in which Gα_q_(123) was replaced by Gα_q_(97). The concentration-dependent response to histamine and the reversibility of the interaction of the two sensor proteins was still given, but the S/B ratio was approx. 3-fold lower (Fig. S3).

It turned out that the quality of D-luciferin, the pH of the surrounding medium and the temperature were critical for the validity of the results. D-luciferin from different suppliers was compared (data not shown) as a substrate of the developed probe, and only those products are listed in the method section, which afforded robust results. When experiments were performed at RT instead at 37 °C or in DMEM, in equilibrium with atmospheric CO_2_ (around 450 ppm), no or only very weak luminescence was detected (data not shown).

As shown in Fig. 2A, onset kinetics of the sensor was slower than expected for the activation of Gα subunits^18,41^. We hypothesize that the observed slowdown is related to the folding of the full-length luciferase (maturation) upon association of the two fragments^22^, thereby neither affecting the Gα_q_/PLC-β3 interaction itself nor downstream signalling. To prove this, we performed a Fura-2 Ca^2+^ assay, affording kinetic information on the transient increase in cytosolic Ca^2+^ with a high temporal resolution. Although the amplitudes of the Ca^2+^ transients were higher in the presence of the sensor (Fig. 2C) – presumably due to differences in cell density and/or by an additional expression of exogenous Gα_q_ and PLC-β3 proteins – the kinetics of intracellular Ca^2+^ mobilisation observed was nearly identical to that obtained from cells expressing the hH_1_R alone (Fig. 2D). Accordingly, the histamine concentration-dependent response was not influenced by the presence of the sensor.

The Gα_q_ inhibitor FR900359 is a valuable tool for analysing signalling pathways involving Gα_q_^3,42^. Therefore, we examined the susceptibility of the modified Gα_q_ to inhibition by FR900359 (Fig. 2E). We were able to inhibit sensor activation stepwise by applying FR900359 at increasing concentrations until the signal was totally abolished at a concentration of 100 nM.

Furthermore, we were interested in the performance of the sensor, when applied in an assay for ligand characterization. With S/B ratios around 5 (Fig. S3) and a Z’ value of 0.7 (Fig. 2F) the new probe should give excellent robust readouts in functional assays.

### Characterization of reference ligands at five different GPCRs

Aiming at a sensor, broadly applicable to diverse Gα_q_-coupled GPCRs, especially to pharmacologically characterize ligands of the respective receptors, we analysed five different GPCRs of the histamine (hH_1_R), muscarinic acetylcholine (hM_1,3,5_R) and neurotensin (hNTS_1_R) family. Our focus lied on literature-described standard agonists in terms of potencies (pEC_50_) and efficacies (E_max_), as well as on antagonists concerning their antagonistic activity (pK_b_). The characterization always included a reference agonist that was able to maximally activate the receptor (defined as 100%). This was either the endogenous agonist or a pharmacologically comparable compound at the respective receptor. All other analysed ligands were normalized regarding their efficacy to the particular reference agonist (Table 1).

**Table 1:** pEC_50_, E_max_ and pK_b_ values of compounds analysed at the hH_1_R, the hM_1,3,5_Rs and the hNTS_1_R.

	compound	pEC_50_	%E_max_	*N*	pK_b_	*N*
hH_1_R	histamine	7.21 ± 0.07	100	8		
	UR-KUM530	8.22 ± 0.04	97.8 ± 3.6	7		
	histaprodifen	6.54 ± 0.07	95.6 ± 1.0	4		
	betahistine	5.95 ± 0.07	98.3 ± 2.1	4		
	mepyramine				8.48 ± 0.07	4
	diphenhydramine				7.36 ± 0.07	5
	cyproheptadine				10.12 ± 0.06	3
	maprotiline				8.74 ± 0.10	4
hM_1_R	carbachol	6.12 ± 0.08	100	4		
	xanomeline	7.19 ± 0.17	80.6 ± 3.2	3		
	oxotremorine	7.32 ± 0.05	83.6 ± 1.8	3		
	iperoxo	9.42 ± 0.05	99.8 ± 2.3	4		
	N′-methyl scopolamine				9.35 ± 0.07	3
	atropine				8.93 ± 0.05	3
	propantheline				9.26 ± 0.05	3
	pirenzepine				7.76 ± 0.05	3
hM_3_R	oxotremorine	7.09 ± 0.09	100	8		
	xanomeline	6.51 ± 0.11	87.2 ± 6.0	5		
	carbachol	6.65 ± 0.06	101.0 ± 4.9	5		
	iperoxo	9.24 ± 0.10	96.4 ± 1.3	4		
	N′-methyl scopolamine				9.34 ± 0.04	5
	atropine				8.69 ± 0.08	5
	propantheline				9.37 ± 0.05	5
	pirenzepine				6.57 ± 0.03	5
hM_5_R	carbachol	6.78 ± 0.06	100	5		
	xanomeline	5.88 ± 0.14	73.3 ± 2.8	4		
	oxotremorine	7.19 ± 0.06	101.4 ± 4.3	4		
	iperoxo	9.80 ± 0.07	101.4 ± 1.1	4		
	N′-methyl scopolamine				9.52 ± 0.08	5
	atropine				8.66 ± 0.05	5
	propantheline				9.82 ± 0.08	5
	pirenzepine				6.65 ± 0.09	5
hNTS_1_R	neurotensin (8–13)	8.79 ± 0.09	100	8		
	SR142948A				8.20 ± 0.06	4

Live HEK293T cells, expressing the developed sensor and the indicated receptor, were investigated regarding their response to standard agonists and antagonist. Data is given as mean ± SEM. *N* denotes the number of biological replicates, each determined in triplicate.

**Figure 3:**
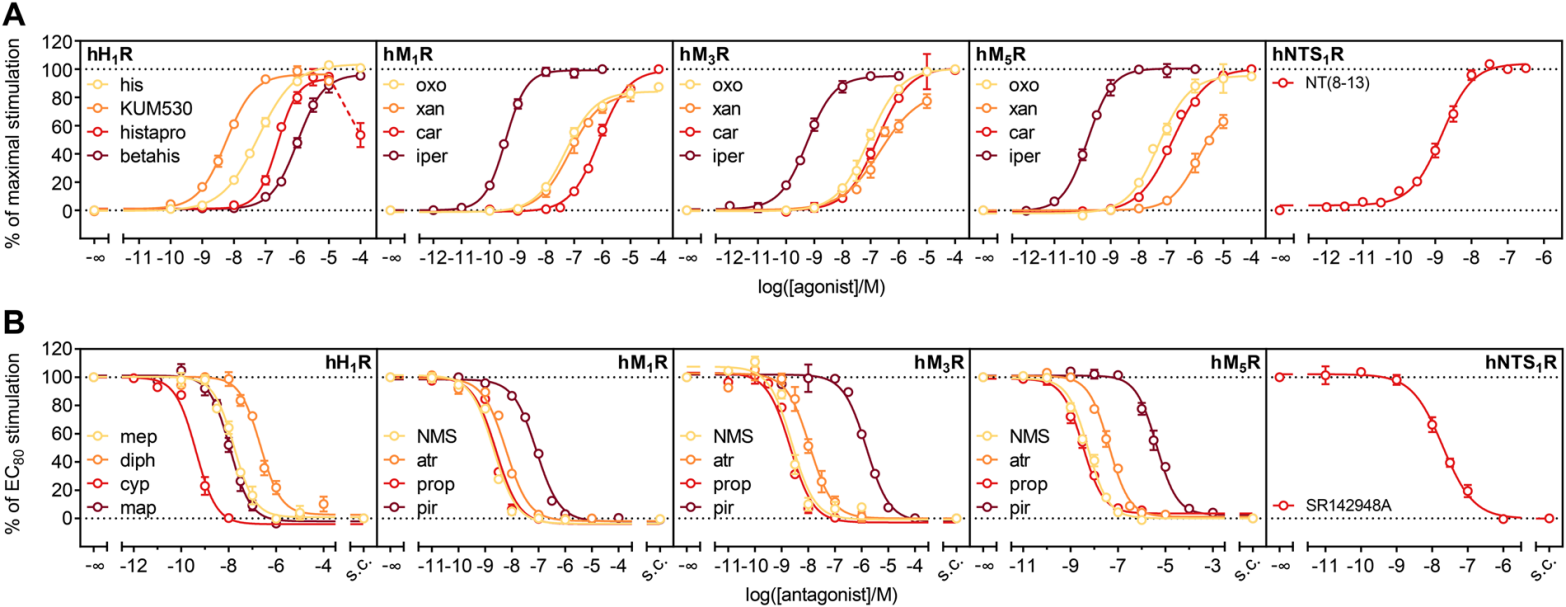
Characterization of standard ligands at the hH_1_R, the hM_1,3,5_R and the hNTS_1_R. Live HEK293T cells, stably expressing the developed sensor and the indicated receptor, were analysed regarding their response to standard agonists (**A**) and antagonists (**B**) for the respective receptors. The substrate D-luciferin was added directly to the cells, and the experiment was carried out at 37 °C. Agonist data was normalized to a reference full agonist for each receptor, maximal stimulation of which was defined as 100% (hH_1_R: histamine, hM_1,5_R: carbachol, hM_3_R: oxotremorine). pEC_50_, E_max_ and pK_b_ values are listed in Table 1 and were in good accordance with data described in literature. Data are presented as means ± SEM of at least three independent experiments, each performed in triplicate. s.c.: solvent control.

At the hH_1_R, the endogenous agonist histamine, the slightly more potent phenylhistamine derivative UR-KUM530, histaprodifen and betahistine, a drug approved for the treatment of Ménière’s disease, were analysed (Fig. 3A). Except for histaprodifen, where luminescence decreased after having reached a plateau, compounds gave robust CRCs. For histaprodifen and derivatives thereof, at higher concentrations toxic effects were reported^3^, which might compromise the luminescence signal. The obtained potencies – with no more than half an order of magnitude difference in EC_50_ – and efficacies were in good agreement with values obtained from the [^32^P]GTPase assay^35,43^ and results from assays addressing alternative signalling pathways, second messengers and holistic methods^3^. The only exception was histaprodifen, which was not always described as a full agonist as in our case^3,16,35,44,45^. Antagonistic activities of the reference ligands mepyramine, diphenhydramine, cyproheptadine and maprotiline also aligned well with values described in literature. The corresponding pK_b_-values differed no more than half a log unit, although especially cyproheptadine was described controversially with at least one and a half orders of magnitude difference in activity across different functional and competition binding assays^3,43,46,47^. For this compound, our data align best with those from competition binding and functional holistic assays^3,46^.

In literature, controversial data were reported for the analysed agonists at the muscarinic receptors, i.e. the ranges of potencies and efficacies for several compounds are very wide (Table S1). The potencies, we determined, fit well into the reported ranges, and, with a few exceptions, the efficacies too. For e.g. xanomeline at the hM_3_R, or oxotremorine at the hM_5_R, different E_max_ values were reported. Although the developed sensor delivers a readout proximal to the receptor, a potential receptor reserve might still influence the observed ligand efficacies^48^, which might be an explanation for the aforementioned discrepancies. This can, of course, also apply to most of the literature reported data. The determined activities (pK_b_ values) of the hM_x_R antagonists were comparable to those obtained from other functional assays or pK_i_ values from radioligand competition binding, with differences not greater than half an order of magnitude^49–56^.

In case of the peptidergic hNTS_1_R, the potency of NT(8–13) was comparable to that determined by BRET between G protein subunits^57^, or in a MAPK-dependent reporter gene assay^58^. The same holds true for the antagonist SR142948A, the pK_b_ of which matches well with values obtained by IP_3_ quantification or radioligand binding^59,60^.

### Live cell luminescence

**Figure 4:**
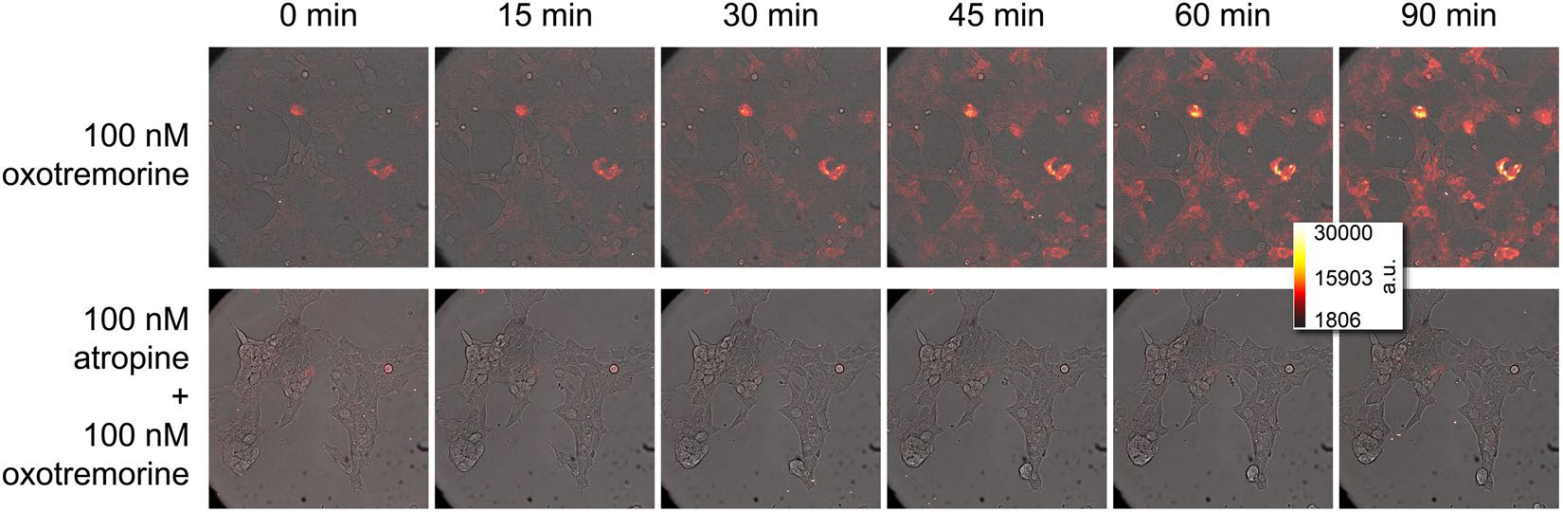
Live cell luminescence imaging of Gα_q_ sensor-expressing HEK293T cells stimulated via the hM_3_R. Shown are the results of one experiment, performed in the agonist (*N* = 3) and the antagonist (*N* = 2) mode, respectively. D-Luciferin was added to the cells before they were transferred to the bioluminescence microscope with its stage warmed to 37 °C. The first frame always shows cells before stimulation. All images were taken with an exposure time of 5 min and are presented as arbitrary light units in false colour. Upon stimulation with 100 nM oxotremorine (approx. EC_60_), a constant saturable increase in luminescence was observed leading to a plateau after approx. 45 min (cf. Fig. S6). No increase was detectable, when the cells were pre-incubated with atropine (100 nM). The supplementary videos clips S1 and S2 show all acquired frames of which only selected are shown here.

To explore the potential of the developed probe in view of future applications, such as multiparametric measurements, e.g. in combination with impedance-based cell sensing, and imaging in laboratory animals, we decided to do live cell bioluminescence microscopy. For this purpose, cells, expressing the hM_3_R and the sensor, were investigated under an inverted microscope equipped with a super-cooled EM-CCD camera. Stimulation of the cells resulted in an increase in luminescence over time, whereas pre-incubation with the antagonist atropine abolished sensor activation completely, as shown in Fig. 4 and Videos S1 and S2. Although confocal resolution was not reached, luminescence was predominantly observed on the edges of the cells, indicating sensor activation associated with the cellular membrane. These observations are in agreement with the fact that both sensor proteins are membrane-associated either due to palmytoylation as in case of Gα_q_ or via hydrophobic regions as in the catalytic core of PLC-β3^40^.

## Conclusion

The SLC approach was applied to the interaction of Gα_q_ and PLC-β3 involved in the signalling cascade of GPCRs. This led, to the best of our knowledge, to the first described SLC-based probe, in which one of the two luciferase fragments was incorporated into the protein sequence of one of the host proteins rather than attached to the termini. As a probe for the Gα_q_/PLC-β3 interaction that makes genetical receptor modifications unnecessary and with its excellent Z’ value of 0.7, the sensor is very suitable for ligand characterization, which was shown for five different GPCRs. Furthermore, the sensor proved to be useful for imaging, as shown for live cell bioluminescence microscopy. Beyond the here described applications the sensor might become a valuable tool for de-orphanization and subsequent determination of signalling pathways of orphan GPCRs, the analysis of Gα_q_ activation in cells endogenously expressing Gα_q_ protein-coupled receptors and imaging in laboratory animals.

## Electronic supplementary material

Supplementary information

Live cell luminescence microscopy – agonist mode

Live cell luminescence microscopy – antagonist mode
