# Supplementary material for: A split luciferase-based probe for quantitative proximal determination of Gαq signalling in live cells
Source: Sci Rep. 2018 Nov 21;8:17179. doi: 10.1038/s41598-018-35615-w (PMC6249299; doi:10.1038/s41598-018-35615-w)
Supplement: Supplementary file 1 — Supplementary information [file 41598_2018_35615_MOESM1_ESM.pdf]

A split luciferase-based probe for quantitative proximal determination of $G\alpha_q$ signalling in live cells

Supplementary information

Timo Littmann[1]*, Takeaki Ozawa[2], Carsten Hoffmann[3], Armin Buschauer[1, †] and Günther Bernhardt[1]*

[1]: Institute of Pharmacy, University of Regensburg, Universitätsstraße 31, D-93053 Regensburg, Germany

[2]: Department of Chemistry, School of Science, University of Tokyo, 7-3-1 Hongo, Bunkyo-ku, Tokyo 113-0033, Japan

[3]: Institute of Molecular Cell Biology, University Hospital Jena, University of Jena, Hans-Knöll-Str. 2, D-07745 Jena, Germany

*: corresponding authors. Institute of Pharmacy, University of Regensburg, Universitätsstraße 31, D-93053 Regensburg, Germany. timo.littmann@ur.de (Timo Littmann); guenther.bernhardt@ur.de (Günther Bernhardt)

†: Deceased, July 18, 2017

Index

Fig. S1: Raw luminescence intensities of all investigated fusion protein combinations. S3

Fig. S2: Anti-Gα_q immunoblot of a lysate from HEK293T cells expressing the developed sensor. S4

Fig. S3: Characterisation of the Gα_q(97) and Gα_q(123) variants in live cells co-expressing the hH_1R. ...S5

Fig. S4: S/B ratios of the sensor when activated by different GPCRs. S6

Fig. S5: Structures of the analysed compounds. S6

Fig. S6: Effect of the oxotremorine concentration on the onset kinetics of the hM_3R-mediated luminescence signal. S7

Table S1: Determined potencies and efficacies of standard agonists at $M_{1,3,5}R$ using the developed probe, in comparison to values reported in literature. S8

Video S1: Live cell luminescence microscopy – agonist mode. S9

Video S2: Live cell luminescence microscopy – antagonist mode. S9

Fig. S7: Original western blot corresponding to Fig. S2. .... S13

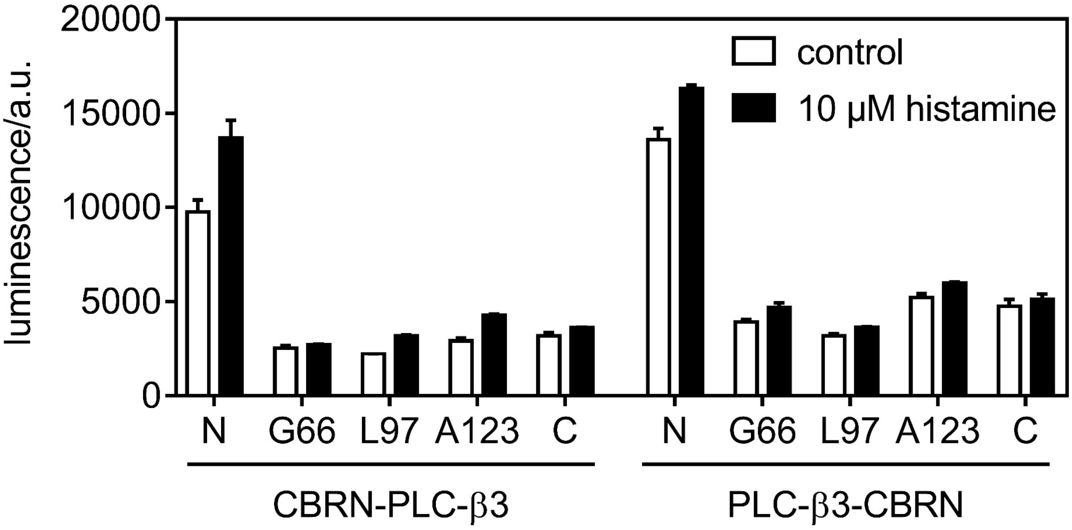

Fig. S1: Raw luminescence intensities of all investigated fusion protein combinations. HEK293T cells were transfected with the different combinations of fusion proteins and the hH_1R. Subsequently, the cells were stimulated with histamine, or with a solvent control for 25 min, before the cells were lysed and the substrate was added. Data (means ± SEM) are shown from one of three experiments, each performed in triplicate.

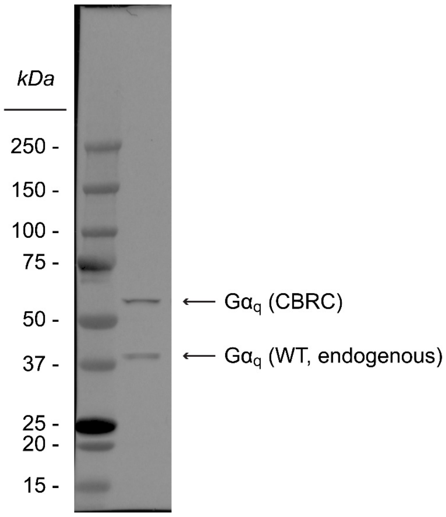

Fig. S2: Anti-Gα_q immunoblot of a lysate from HEK293T cells stably expressing the developed sensor. To confirm reliable cleavage of the 2A autoproteolysis site separating CBRN-PLC-β3 and Gα_q(123) an immunoblot with an anti-Gα_q antibody was performed. The detection revealed two distinct bands originating from endogenous Gα_q (≈ 42 kDa) and most probably from the Gα_q CBRC fusion protein (≈ 59 kDa). No higher molecular weight protein was stained, indicating complete cleavage of the autoproteolysis site. It can also be seen that expression of the modified Gα_q was comparable with endogenous Gα_q. Blotting was performed as follows: HEK293T cells with the pIRESpuro3 CBRN-PLC-β3-2A-Gα_q(123) vector stably integrated in their genome were lysed using a RIPA lysis buffer (50 mM Tris, 0.1% sodium dodecyl sulfate, 0.5% sodium deoxycholate, 1% Triton X 100, 150 mM NaCl) supplemented with a SIGMAFAST protease inhibitor cocktail (Sigma, Germany). Of this lysate, 20 µg were separated on a 8-16% Novex Tris-Glycine polyacrylamide gel (Thermo Scientific, Germany). The proteins were blotted on a nitrocellulose membrane at 0.13 A for 1 h, and unspecific binding sites were blocked using skim milk powder (5%) in phosphate-buffered saline (137 mM NaCl, 2.7 mM KCl, 10 mM Na_2HPO_4, 1.8 mM KH_2PO_4), supplemented with Tween 20 (0.05%) (PBS-T), for 1 h. The polyclonal primary antibody against Gα_q, produced in rabbit (Cat. 371754, Merck Millipore, Germany), was used at a dilution of 1:500 in PBS-T with milk powder (5%) to incubate the blot at 4 °C overnight. On the next day, the blot was washed three times using PBS-T and a HRP-conjugated secondary antibody against rabbit IgG, produced in donkey (sc-2313, Santa Cruz, TX, USA), was added at a dilution of 1:10000 in PBS-T devoid of milk powder. The blot was incubated with the secondary antibody for 1 h before being developed using an ECL reagent (Bio-Rad, Germany). Luminescence emitted by the stained bands was quantified using a ChemiDoc MP imaging system (Bio-Rad, Germany) with an exposure time of 2 min. Shown is a superposition of the chemiluminescent blot with a colorimetric image showing the molecular weight marker Precision Plus Dual Color (Bio-Rad). The image shows the full length of the blot. The corresponding original western blot is shown in Fig. S7.

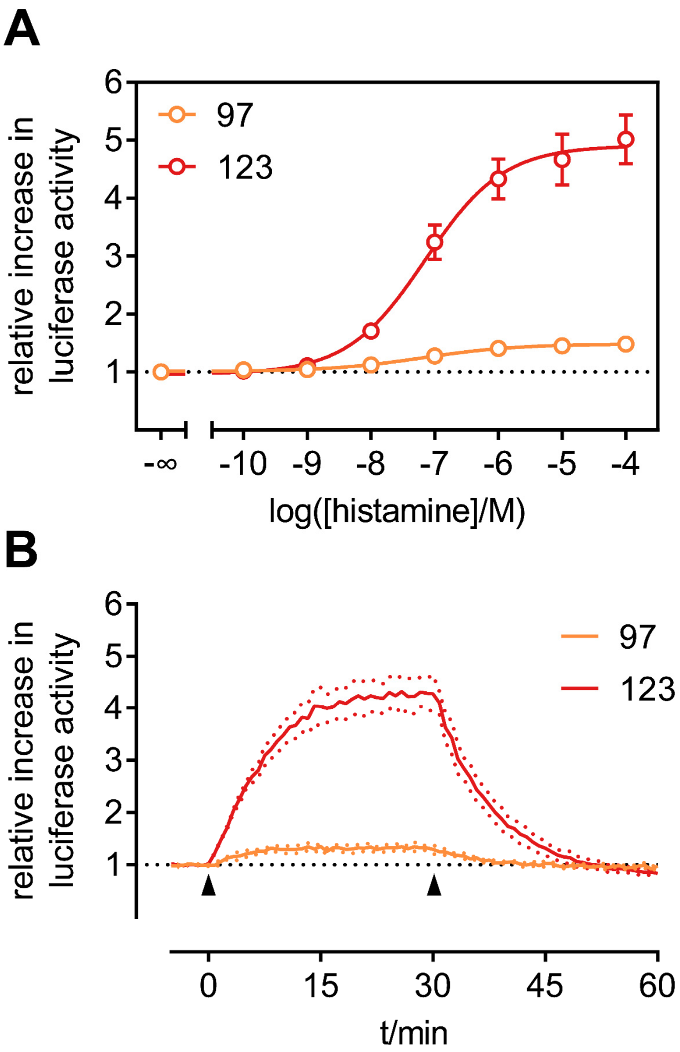

Fig. S3: Characterisation of the Gα$_q$(97) and Gα$_q$(123) variants in live cells co-expressing the hH$_1$R. HEK293T cells, stably transfected either with the pIRESpuro3 CBRN-PLC-β3-2A-Gα$_q$(97) or the pIRESpuro3 CBRN-PLC-β3-2A-Gα$_q$(123) together with the hH$_1$R, were generated. **A**: The cells were analysed with respect to their response to increasing concentrations of histamine, yielding a large difference in signal-to-background (S/B) ratio (Gα$_q$(97): 1.49 ± 0.08, Gα$_q$(123): 5.02 ± 0.42), but similar pEC$_{50}$ values for histamine (Gα$_q$(97): 7.18 ± 0.22, Gα$_q$(123): 7.15 ± 0.16). **B**: The cells were stimulated with 300 nM histamine (first arrow) before mepyramine to a final concentration of 1 µM was added (second arrow). Again, the Gα$_q$(97) variant shows a lower S/B ratio, but the interaction of both sensor protein pairs is fully reversible. Data represents means ± SEM from three independent experiments, performed in triplicate.

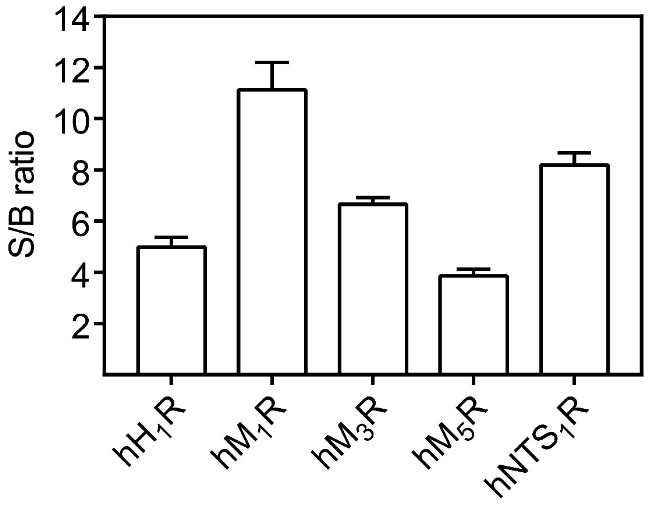

Fig. S4: S/B ratios of the sensor when activated by different GPCRs. HEK293T cells, expressing the developed sensor were stimulated via the given receptor. Luminescence intensities obtained from maximally stimulated cells were divided by those obtained from unstimulated cells. Data represents means ± SEM of at least three independent experiments, each performed in triplicate.

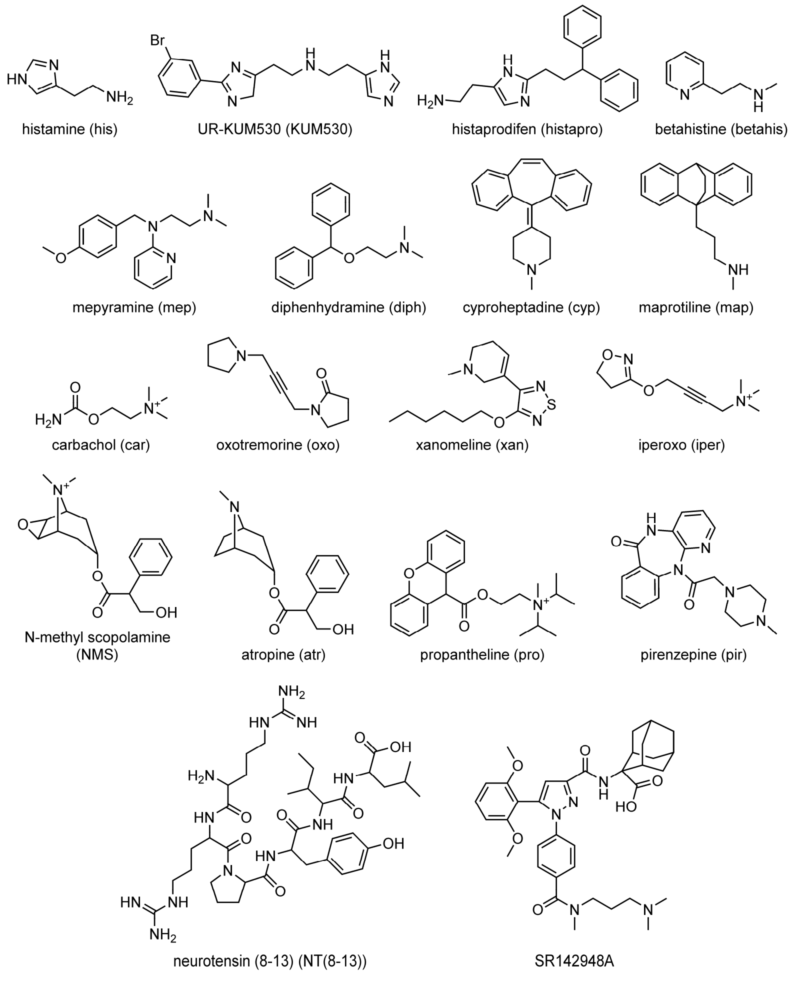

Fig. S5: Structures of the analysed compounds.

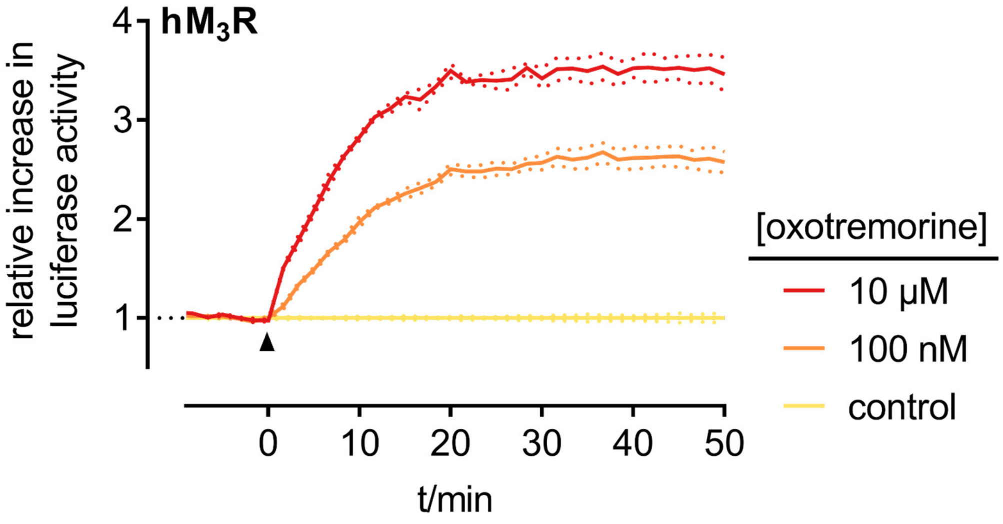

Fig. S6: Effect of the oxotremorine concentration on the onset kinetics of the hM_3R-mediated luminescence signal. The cells were stimulated with a concentration at E_{max} (10 µM) in comparison to the concentration used in the imaging experiments (100 nM, approx. EC_{60}). Data are given as means ± SEM and are a representative of eight independent experiments, each performed in triplicate.

Table S1: Determined potencies and efficacies of standard agonists at $M_{1,3,5}R$ using the developed probe, in comparison to values reported in literature. Live HEK293T cells, expressing the developed sensor and the indicated receptor, were investigated with respect to their response to standard agonists. Data are given as means ± SEM. *N* denotes the number of biological replicates, each determined in triplicate. Except for [17] were ex vivo (rabbit and guinea pig) pharmacology results are reported, all other references contain in vitro data obtained at human receptors.

					**Competition binding**	**Proximal readout assays (e.g. [^{35}S]-GTPγS)**		**Second messenger assays (e.g. IP_x, $[Ca^{2+}]_i$)**		**Distal readout assays (e.g. gene transcription, ex vivo)**	
	**compound**	**pEC_{50}**	**$\%E_{max}$**	***N***	**pK_i**	**pEC_{50}**	**$\%E_{max}$**	**pEC_{50}**	**$\%E_{max}$**	**pEC_{50}**	**$\%E_{max}$**
**hM_1R**	carbachol	6.12 ± 0.08	100	4	3.17 – 4.46[1-3]	4.67 – 6.08[4-6]	93 – 100[4-6]	4.73 – 6.96[6-12]	99.6 – 103[6-12]	5.19 – 5.82[3,7,13,14]	93.6 – 100[3,7,13,14]
	xanomeline	7.19 ± 0.17	80.6 ± 3.2	3	6.68 ± 0.02[3]	5.98 – 8.15[4,15]	40 – 127[4,15]	6.96 – 7.78[7,9,10,16]	29.8 – 117[7,9,10,16]	6.82 – 8.34[3,7]	41.7 – 105[3,7]
	oxotremorine	7.32 ± 0.05	83.6 ± 1.8	3	5.48 – 5.86[1,2]	6.64 ± 0.21[6]	62 ± 4[6]	5.70 – 6.70[6,12]	51 – 56[6,12]	6.41 – 7.72[13,17]	75 – 100[13,17]
	iperoxo	9.42 ± 0.05	99.8 ± 2.3	4		> 7[18]	100[18]	7.97 ± 0.09[16]	101 ± 3.3[16]	8.69 – 9.87[17,19]	100 – 102[17,19]
**hM_3R**	oxotremorine	7.09 ± 0.09	100	8	5.28 – 5.71[1,2]			6.39 – 7.33[10,12]	48 – 100[10,12]	6.68 – 7.98[13,17]	100[13,17]
	xanomeline	6.51 ± 0.11	87.2 ± 6.0	5	7.21 ± 0.06[3]			7.10 ± 0.10[10]	≈ 106[10]	6.16 – 6.82[3,14]	97.5 – 100[3,14]
	carbachol	6.65 ± 0.06	101 ± 4.9	5	3.61 – 4.42[1-3]	5.83 – 6.3[5,20]	100[5,20]	5.33 – 7.40[10-12,20,21]	84 – 131[10-12,20,21]	5.85 – 6.96[3,13]	91 – 100[3,13]
	iperoxo	9.24 ± 0.10	96.4 ± 1.3	4						9.78 ± 0.10[17]	100[17]
**hM_5R**	carbachol	6.78 ± 0.06	100	5	4.51 – 4.92[1,3]			5.72 – 6.9[10,12,21-23 24]	100[10,12,21,22 24]	5.89 – 7.22[13,14,25]	100[13,14,25]
	xanomeline	5.88 ± 0.14	73.3 ± 2.8	4	7.09 ± 0.19[3]			6.52 – 7.63[10,21,22 24]	25 – 80[10,21,22 24]		
	oxotremorine	7.19 ± 0.06	101.4 ± 4.3	4	6.05 ± 0.04[1]			6.24 – 7.29[10,12]	58 – 88[10,12]	7.26[13]	74 ± 2[13]
	iperoxo	9.80 ± 0.07	101.4 ± 1.1	4							

Video S1: Live cell luminescence microscopy – agonist mode. HEK293T cells expressing the developed sensor and the hM_3R were supplemented with D-luciferin and were transferred to a bioluminescence microscope with its stage warmed to 37 °C. The first frame always shows cells before stimulation. All images were taken with an exposure time of 5 min and are presented false-coloured (the scale bar representing the intensities in arbitrary light units can be found in Fig. 4). Stimulation was realized using oxotremorine (100 nM). A constant saturable increase can be observed leading to a plateau approx. after 45 min

Video S2: Live cell luminescence microscopy – antagonist mode. The experiment was essentially described as for Video S1 with the exception that atropine (100 nM) was added prior to the very first frame. Atropine blocked the receptors and abolishes the oxotremorine-promoted effect observed in Video S1.

References

1 Dong, G. Z., Kameyama, K., Rinken, A. & Haga, T. Ligand binding properties of muscarinic acetylcholine receptor subtypes (m1-m5) expressed in baculovirus-infected insect cells. *J Pharmacol Exp Ther* **274**, 378-384 (1995).

2 Jakubík, J., Bačáková, L., El-Fakahany, E. E. & Tuček, S. Positive cooperativity of acetylcholine and other agonists with allosteric ligands on muscarinic acetylcholine receptors. *Mol Pharmacol* **52**, 172-179 (1997).

3 Wood, M. D. *et al.* Functional comparison of muscarinic partial agonists at muscarinic receptor subtypes hM_1, hM_2, hM_3, hM_4 and hM_5 using microphysiometry. *Br J Pharmacol* **126**, 1620-1624, doi:10.1038/sj.bjp.0702463 (1999).

4 Jakubík, J., El-Fakahany, E. E. & Doležal, V. Differences in kinetics of xanomeline binding and selectivity of activation of G proteins at M_1 and M_2 muscarinic acetylcholine receptors. *Mol Pharmacol* **70**, 656-666, doi:10.1124/mol.106.023762 (2006).

5 Jakubík, J., Janíčková, H., Randáková, A., El-Fakahany, E. E. & Doležal, V. Subtype differences in pre-coupling of muscarinic acetylcholine receptors. *PLoS One* **6**, e27732, doi:10.1371/journal.pone.0027732 (2011).

6 Waelbroeck, M. Activation of guanosine 5'-[γ-^{35}S]thio-triphosphate binding through M_1 muscarinic receptors in transfected Chinese Hamster ovary cell membranes: 2. Testing the "two-states" model of receptor activation. *Mol Pharmacol* **59**, 886-893 (2001).

7 Christopoulos, A., Pierce, T. L., Sorman, J. L. & El-Fakahany, E. E. On the unique binding and activating properties of xanomeline at the M_1 muscarinic acetylcholine receptor. *Mol Pharmacol* **53**, 1120-1130 (1998).

8 Digby, G. J. *et al.* Novel allosteric agonists of M_1 muscarinic acetylcholine receptors induce brain region-specific responses that correspond with behavioral effects in animal models. *J Neurosci* **32**, 8532-8544, doi:10.1523/JNEUROSCI.0337-12.2012 (2012).

9 Randáková, A. *et al.* Classical and atypical agonists activate M_1 muscarinic acetylcholine receptors through common mechanisms. *Pharmacol Res* **97**, 27-39, doi:10.1016/j.phrs.2015.04.002 (2015).

10 Šantrůčková, E., Doležal, V., El-Fakahany, E. E. & Jakubík, J. Long-term activation upon brief exposure to xanomleline is unique to M_1 and M_4 subtypes of muscarinic acetylcholine receptors. *PLoS One* **9**, e88910, doi:10.1371/journal.pone.0088910 (2014).

11 Pronin, A. N., Wang, Q. & Slepak, V. Z. Teaching an Old Drug New Tricks: Agonism, Antagonism, and Biased Signaling of Pilocarpine through M_3 Muscarinic Acetylcholine Receptor. *Mol Pharmacol* **92**, 601-612, doi:10.1124/mol.117.109678 (2017).

12 Kukkonen, J. P., Nasman, J., Ojala, P., Oker-Blom, C. & Akerman, K. E. Functional properties of muscarinic receptor subtypes Hm1, Hm3 and Hm5 expressed in Sf9 cells using the baculovirus expression system. *J Pharmacol Exp Ther* **279**, 593-601 (1996).

13 Bräuner-Osborne, H. & Brann, M. R. Pharmacology of muscarinic acetylcholine receptor subtypes (m1-m5): high throughput assays in mammalian cells. *Eur J Pharmacol* **295**, 93-102 (1996).

14 Burstein, E. S., Spalding, T. A. & Brann, M. R. Pharmacology of muscarinic receptor subtypes constitutively activated by G proteins. *Mol Pharmacol* **51**, 312-319 (1997).

15 Salah-Uddin, H. *et al.* Pharmacological assessment of m1 muscarinic acetylcholine receptor-gq/11 protein coupling in membranes prepared from postmortem human brain tissue. *J Pharmacol Exp Ther* **325**, 869-874, doi:10.1124/jpet.108.137968 (2008).

16 van der Westhuizen, E. T. *et al.* Assessment of the Molecular Mechanisms of Action of Novel 4-Phenylpyridine-2-One and 6-Phenylpyrimidin-4-One Allosteric Modulators at the M_1 Muscarinic Acetylcholine Receptors. *Mol Pharmacol* **94**, 770-783, doi:10.1124/mol.118.111633 (2018).

17 Dallanoce, C. *et al.* Synthesis and functional characterization of novel derivatives related to oxotremorine and oxotremorine-M. *Bioorg Med Chem* **7**, 1539-1547 (1999).

18 Messerer, R. *et al.* FRET Studies of Quinolone-Based Bitopic Ligands and Their Structural Analogues at the Muscarinic M_1 Receptor. *ACS Chem Biol* **12**, 833-843, doi:10.1021/acschembio.6b00828 (2017).

19 Chen, X. *et al.* Rational design of partial agonists for the muscarinic M_1 acetylcholine receptor. *J Med Chem* **58**, 560-576, doi:10.1021/jm500860w (2015).

20 Noetzel, M. J., Grant, M. K. & El-Fakahany, E. E. Mechanisms of M3 muscarinic receptor regulation by wash-resistant xanomeline binding. *Pharmacology* **83**, 301-317, doi:10.1159/000214843 (2009).

21 Randáková, A., Rudajev, V., Doležal, V., Boulos, J. & Jakubík, J. Novel long-acting antagonists of muscarinic ACh receptors. *Br J Pharmacol* **175**, 1731-1743, doi:10.1111/bph.14187 (2018).

22 Randáková, A. *et al.* Role of membrane cholesterol in differential sensitivity of muscarinic receptor subtypes to persistently bound xanomeline. *Neuropharmacology* **133**, 129-144, doi:10.1016/j.neuropharm.2018.01.027 (2018).

23 Berizzi, A. E. *et al.* Molecular Mechanisms of Action of M5 Muscarinic Acetylcholine Receptor Allosteric Modulators. *Mol Pharmacol* **90**, 427-436, doi:10.1124/mol.116.104182 (2016).

24 Grant, M. K. & El-Fakahany, E. E. Persistent binding and functional antagonism by xanomeline at the muscarinic M5 receptor. *J Pharmacol Exp Ther* **315**, 313-319, doi:10.1124/jpet.105.090134 (2005).

25 Bräuner-Osborne, H., Ebert, B., Brann, M. R., Falch, E. & Krogsgaard-Larsen, P. Functional partial agonism at cloned human muscarinic acetylcholine receptors. *Eur J Pharmacol* **313**, 145-150 (1996).

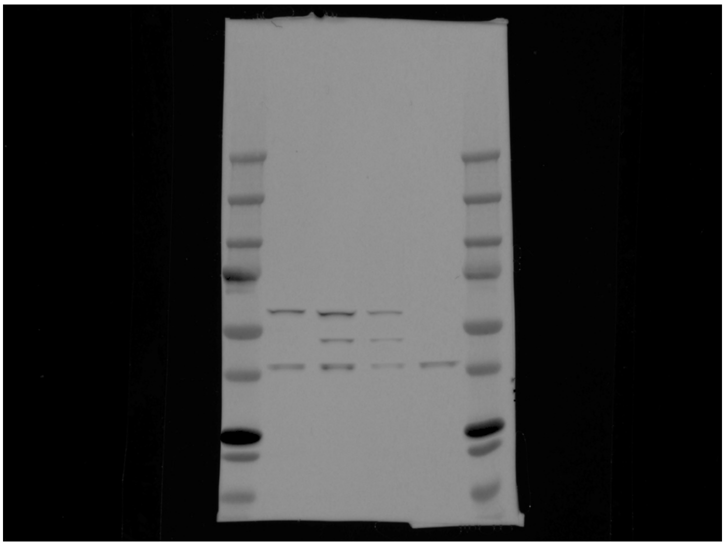

Fig. S7: Original western blot corresponding to Fig. S2. The relevant lanes, shown in Fig. S2, are the left lane containing the weight standard and the lane next to it.
